# The health impact of long COVID: a cross-sectional examination of health-related quality of life, disability, and health status among individuals with self-reported post-acute sequelae of SARS CoV-2 infection at various points of recovery

**DOI:** 10.1186/s41687-023-00572-0

**Published:** 2023-03-21

**Authors:** Casey R. Tak

**Affiliations:** Department of Pharmacotherapy, College of Pharmacy, University of Utah, Salt Lake City, UT USA

## Abstract

**Objective:**

The novel Coronavirus (COVID-19) has continued to present a significant burden to global public health efforts. The purpose of this study was to estimate the health-related quality of life, disability, and health status of individuals with self-reported long COVID at various lengths of recovery.

**Methods:**

We conducted a cross-sectional online survey of individuals with self-reported long COVID. Participants were asked to complete the five-item EuroQOL EQ-5D-5L and EQ visual analog scale, the 12-item World Health Organization Disability Assessment Schedule (WHODAS) 2.0 and the 10-item Patient Reported Outcome Measurement Information System (PROMIS) Global Health v1.2 short form. Descriptive and inferential statistics were used to characterize the responses and differences across groups.

**Results:**

Eighty-two participants from 13 countries completed the EQ-5D-5L, 73 completed the WHODAS 2.0 and 80 participants completed the PROMIS. The mean EQ-5D-5L utility score was 0.51. The mean WHODAS score was 49.0. In the previous 30 days, participants reported their symptoms affected them for a mean of 24 days, they were totally unable to carry out usual activities for 15 days, and they cut back or reduced activities for 26 days. The mean PROMIS physical health and mental health scores were 10.7 and 8.6, respectively, corresponding to below-average health. No significant differences were detected across time or according to severity of acute infection.

**Conclusions:**

Long COVID presents a significant chronic health burden to adults in the US and abroad. This health burden may persist for many months post-acute infection.

**Supplementary Information:**

The online version contains supplementary material available at 10.1186/s41687-023-00572-0.

## Highlights

1. Individuals with long COVID self-report persistently low levels of health-related quality of life, high levels of disability, and low levels of physical and mental health, many months post infection.
2. There was no significant relationship detected between the self-reported severity of acute infection and the self-reported severity of long COVID symptoms.

**Supplementary Information:**

The online version contains supplementary material available at 10.1186/s41687-023-00572-0.

## Introduction

The novel Coronavirus (COVID-19) has continued to present a significant burden to global public health efforts with, as of October 2022, over 620 million cases worldwide [1]. Although most patients fully recover from the acute infection, a significant number of individuals continue to experience long-term illness, known as post-acute sequelae of SARS CoV-2 infection (PASC) or long COVID.

Long COVID, defined by the World Health Organization (WHO) as a condition occurring at least three months post onset of a probable or confirmed COVID-19 infection and lasting for at least two months that is otherwise inexplicable, estimates to impact 10–30% of COVID-19 patients, although estimates vary [2]. The health consequences of long COVID are substantial and can affect many organ systems; over 200 specific symptoms have been associated with long COVID [3, 4]. Moreover, previous reported have detailed the impact that long COVID on employment and activities of daily living (ADL). Individuals with long COVID have 23% higher odds of being unemployed and, among those who do work, a 21% lower odds of working full-time; further, studies have found reductions in performing ADL [5, 6].

Previous studies have estimated the health-related quality of life (HRQOL) in patients who were previously hospitalized with COVID-19 and continued to experience symptoms [7]. However, to date, the impact of long COVID on HRQOL, disability, and health status has not been thoroughly evaluated among individuals who were not hospitalized with an acute COVID-19 infection and among those who have continued experiencing symptoms many months past the initial infection [7]. The purpose of this study was to estimate the HRQOL, disability, and health status of a broader group of individuals with self-reported long COVID at various lengths of recovery.

## Methods

We recruited participants from the social media platforms Reddit and Facebook who were subscribed to or following recovery/support groups related to long COVID to participate in a cross-sectional survey. We included adults aged 18+ who had self-reported long COVID, defined at the time by the Centers for Disease Control and Prevention (CDC) as having symptoms that continue for at least four weeks beyond the acute COVID infection [8]. Because the symptoms of long COVID may vary considerably from person to person and not all long COVID patients received a COVID test, we did not restrict entry to any particular symptoms or to those with a positive test [9]. Prior to beginning the survey, potential participants were provided with a cover letter consent form with information on the study. After reviewing the information about the study, individuals were given the option to provide written informed consent and continue with the study or discontinue. Individuals who provided informed consent were documented in the survey database. Because of the mental fatigue that many patients with long COVID experience, the survey tool was designed to allow participants to save their data and return later to complete the questionnaire [9]. Individuals who continued to participate indicated whether they were tested for COVID and whether they were tested for antibodies. Those who responded affirmatively to either test were considered “COVID confirmed”; the remainder were considered “COVID suspected.” Participants reported time since onset of long COVID symptoms. Responses were collected in October and November 2021.

Questions used in the survey were informed and piloted by individuals with self-reported long COVID (see “Additional file 1: Appendix” for full survey instrument). Demographic (age, race, ethnicity, employment status, educational attainment) and clinical (e.g., previous diagnoses, current or past long COVID symptoms) characteristics were collected. Participants were asked to complete the five-item EuroQOL EQ-5D-5L to estimate HRQOL, the EQ visual analog scale (VAS), the 12-item World Health Organization Disability Assessment Schedule (WHODAS) 2.0 and the 10-item Patient Reported Outcome Measurement Information System (PROMIS) Global Health v1.2 short form. Scores from the EQ-5D-5L were translated to a utility score using the US valuation, where “1” represents perfect health and “0” represents a health condition equivalent to death [10]. The single score from the EQ VAS, which asks participants to choose a single score between 0 and 100 where 0 represent the worst imaginable health and 100 represents the best imaginable health, was captured [10]. Scores from the WHODAS 2.0 were summed and transformed to a 0–100 scale, with “0” representing no disability and “100” representing full disability [11]. Responses from PROMIS were used to calculate a physical health (PH) and mental health (MH) score on a scale from 4 to 20, with higher scores indicating better health [12, 13]. The PH and MH scores were also used to generate a T-score using the HealthMeasures Scoring Service [12]. A t-score of 50 represents the average score in a reference population with scores above 50 indicating better health and scores below 50 representing poorer health. The cutoffs for PH and MH were as follows, respectively: Excellent: 58 and 56; Very Good: 50 and 48; Good: 42 and 40; Fair: 35 and 29. Scores below “Fair” were considered “Poor” health.

We also collected information on factors hypothesized to be associated with long COVID, such as severity of acute COVID-19 illness, receipt of COVID-19 vaccination, symptoms experienced, self-reported presence of at least one episode of post-exertional malaise (PEM, as defined by CDC as “the worsening of symptoms following even minor physical or mental exertion, with symptoms typically worsening 12–48 h after activity and lasting for days or even weeks” [14]), body mass index (BMI), pre-existing health conditions, and demographics characteristics such as age and gender. [15]

Descriptive statistics summarized the respondent characteristics and their survey responses. EQ-5D-5L utility, WHODAS 2.0 scores, and PROMIS PH and MH scores were summarized and stratified across respondent characteristics to assess for trends. Mann–Whitney U tests and Kruskal–Wallis tests were used to determine statistical significance, as appropriate. Study data were collected and managed using REDCap electronic data capture tools hosted at the University of Utah [16]. Data were analyzed in SAS v9.4 (SAS Institute, Cary, NC). Violin plots, which show a smoothed data density along a boxplot and allow a deeper exploration of the data and their distribution, were created in R v4.1.1 (R Core Team, Vienna, Austria) [17, 18]. The University of Utah Institutional Review Board reviewed this study and deemed it exempt.

## Results

Participant details are outlined in Table 1. Eighty-two participants from 13 countries (US [n = 42], United Kingdom [n = 17], other European countries [n = 9], Canada [n = 4], other/missing [n = 10]) completed the EQ-5D-5L, 73 completed the WHODAS 2.0 and 80 participants completed the PROMIS. The average (median) age was 37 (40), 60% were women, 86% were white, 73% had completed at least four years of higher education, and 77% lived in self-reported urban or suburban areas. The mean (median) BMI was 25.5 (24.6) and the mean (median) minutes of exercise per week pre-COVID was 333 (205). Over half (60%) of individuals indicated having either a positive COVID test or a positive antibody test (COVID confirmed). About half of respondents (48%) indicated having either mild symptoms (i.e., did not seek medical attention) or were asymptomatic for the initial acute COVID infection whereas the remainder indicated having moderate (i.e., sought some medical attention but was not hospitalized) or severe (i.e., hospitalized for COVID) symptoms. Most (87%) had received at least one COVID vaccine.

**Table 1:** EQ-5D-5L utility values, WHODAS scores, and PROMIS scores stratified by select demographic and clinical characteristics

Variable	N (%)	Median (IQR) utility value (n = 82)	Median (IQR) WHODAS score (n = 73)	Median (IQR) PROMIS PH score (n = 80)	Median (IQR) PROMIS MH score (n = 77)
Total	82 (100)	0.59 (0.42)	50.0 (27.1)	10.5 (4.0)	8.0 (4.0)
*Time since onset of infection/symptoms*					
0–6 months	15 (18.3)	0.62 (0.81)	41.7 (31.3)	12.0 (6.0)	6.0 (5.0)
7–12 months	20 (24.4)	0.59 (0.31)	44.8 (25.0)	9.0 (4.0)	8.0 (6.0)
13–18 months	14 (17.1)	0.48 (0.35)	62.5 (20.9)	10.0 (3.0)	8.0 (5.0)
19+ months	20 (24.4)	0.59 (0.33)	53.2 (16.6)	11.0 (3.0)	8.0 (3.0)
Unknown/Missing*	13 (15.9)	0.62 (0.39)	47.9 (41.7)	11.5 (6.0)	9.5 (5.0)
*Severity of acute COVID-19 infection*					
Asymptomatic	4 (4.9)	0.33 (0.56)	58.4 (30.3)	8.0 (3.0)	7.0 (4.5)
Mild	35 (42.7)	0.62 (0.51)	45.9 (33.3)	12.0 (5.0)	8.0 (5.5)
Moderate	36 (43.9)	0.53 (0.33)	52.1 (25.0)	10.0 (3.5)	8.0 (4.0)
Severe	3 (3.7)	0.60 (0.19)	37.5 (14.6)	11.0 (3.0)	9.0 (6.0)
Unknown/missing*	4 (4.9)	0.74 (0.29)	47.9 (39.5)	12.0 (2.0)	13.0 (6.0)
*Gender*					
Women	44 (53.7)	0.59 (0.32)	52.1 (27.1)^†^	10.0 (4.0)^†^	8.5 (4.0)
Men	30 (36.6)	0.59 (0.50)	42.8 (33.4)^†^	12.0 (4.0)^†^	6.5 (5.0)
Unknown/missing*	8 (9.8)	0.60 (0.67)	47.9 (33.3)	10.0 (5.0)	8.0 (5.0)
*Age*					
18–29	21 (25.3)	0.36 (0.53)	47.9 (31.3)	11.0 (5.0)	6.0 (5.5)
30–49	31 (37.4)	0.62 (0.19)	51.1 (25.0)	11.0 (3.0)	8.0 (3.0)
50+	19 (22.9)	0.59 (0.38)	50.0 (31.3)	9.0 (4.0)	9.0 (5.0)
Unknown/missing*	11 (14.5)	0.54 (0.67)	50.0 (12.5)	9.5 (5.0)	7.0 (4.0)
*Education status*					
Less than Bachelor’s Degree	20 (24.4)	0.64 (0.30)	43.8 (27.1)	12.0 (6.0)	6.0 (4.0)
Bachelor’s Degree	35 (42.7)	0.56 (0.31)	52.1 (27.1)	10.0 (4.0)	8.5 (4.0)
Master’s or Doctorate	19 (23.2)	0.59 (0.50)	52.1 (33.3)	10.0 (4.0)	7.5 (5.0)
Unknown/Missing*	8 (9.8)	0.60 (0.67)	47.9 (33.3)	10.0 (5.0)	8.0 (5.0)
*Geographic setting*					
Urban/suburban	57 (69.5)	0.56 (0.39)	52.1 (29.2)	10.5 (4.5)	8.0 (5.0)
Rural	15 (18.3)	0.60 (0.39)	51.1 (22.9)	10.0 (4.0)	8.0 (2.0)
Unknown/missing*	10 (12.2)	0.69 (0.67)	47.9 (29.1)	11.0 (4.0)	8.0 (4.0)
*Insurance*					
Private	23 (28.1)	0.52 (0.35)	47.9 (20.9)	10.0 (3.0)	8.0 (5.0)
Government	17 (20.7)	0.56 (0.22)	53.2 (21.9)	11.0 (4.0)	8.0 (2.5)
Other Insurance	2 (2.4)	0.68 (0.19)	31.3 (33.3)	10.5 (7.0)	13.5 (5.0)
Uninsured	10 (12.2)	0.68 (0.22)	45.9 (29.2)	10.5 (5.0)	8.5 (3.0)
Unknown/missing*	33 (40.2)	0.60 (0.58)	51.1 (37.5)	11.0 (5.0)	7.5 (4.5)
*BMI*					
< 25	35 (42.7)	0.56 (0.60)	47.9 (31.3)	10.0 (4.0)	8.0 (5.0)
25 to < 30	13 (15.7)	0.58 (0.29)	46.9 (20.9)	12.0 (5.0)	8.0 (1.0)
30+	15 (18.1)	0.59 (0.32)	47.9 (20.8)	11.0 (5.0)	8.0 (5.0)
Unknown/missing*	19 (24.1)	0.63 (0.37)	52.1 (2.1)	10.0 (3.0)	8.0 (6.0)
*Exercise per week (pre-COVID)*					
0 to < 2 h	19 (23.2)	0.59 (0.26)^†^	43.8 (23.0)	11.0 (5.0)	8.0 (4.0)
2 to < 5 h	26 (31.7)	0.63 (0.43)^†^	47.9 (27.1)	11.0 (2.0)	8.0 (5.0)
5+ hours	31 (37.8)	0.41 (0.51)^†^	56.3 (24.0)	9.0 (4.0)	8.0 (5.0)
Unknown/missing*	6 (7.3)	0.76 (0.34)	35.5 (33.3)	11.0 (4.0)	8.0 (3.0)
*COVID status*					
Confirmed	49 (59.8)	0.56 (0.45)	47.9 (25.0)	11.0 (5.0)	8.0 (5.0)
Suspected	33 (40.2)	0.59 (0.33)	52.1 (28.2)	10.0 (3.0)	9.0 (4.0)
*Received COVID vaccine*					
Yes	71 (86.6)	0.58 (0.40)	52.1 (27.1)	10.0 (4.0)	8.0 (4.0)
No	10 (12.2)	0.63 (0.50)	41.7 (14.6)	11.5 (5.0)	7.5 (6.0)
*Symptom count (previous 30 days)*					
< 10	36 (43.9)	0.61 (0.40)^†^	41.7 (27.1)^†^	11.0 (4.0)^†^	8.0 (6.0)
10–29	27 (32.9)	0.68 (0.43)^†^	43.8 (18.8)^†^	12.0 (4.0)^†^	8.0 (4.0)
30+	19 (23.2)	0.44 (0.31)^†^	62.5 (18.7)^†^	8.0 (3.0)^†^	8.0 (3.0)
*Long COVID clinic*					
Yes	14 (18.4)	0.29 (0.65)^†^	49.0 (29.2)	9.0 (3.0)^†^	6.5 (3.0)^†^
No	58 (76.3)	0.59 (0.39)^†^	56.3 (27.0)	11.0 (4.0)^†^	8.0 (5.0)^†^
*Medication use specific to long COVID*					
Yes	47 (57.3)	0.54 (0.46)	53.2 (25.0)^†^	10.0 (4.0)	8.0 (5.0)
No	25 (30.5)	0.59 (0.31)	43.8 (25.0)^†^	11.0 (5.0)	9.0 (5.0)
Unsure*	3 (3.7)	0.41 (0.24)	35.4 (14.6)	12.0 (3.0)	9.0 (10.0)
Unknown/missing*	7 (8.5)	0.78 (0.39)	35.4 (18.7)	11.0 (4.0)	–

Utility values range from “0”, respresenting a health state equivalent to death, and “1”, representing “perfect health.” WHODAS scores range from “0” representing no disability and “100” representing full disability. PROMIS PH and MH Scores range from “4” to “20” with higher scores indicative of better health

*PH* physical health, *MH* mental health, *BMI* body mass index

*Unknown/missing/unsure categories were not used in the calculation of *p* values

^†^Significant at *p* < 0.05

Respondents began experiencing long COVID symptoms on average 13 months (sd: 6.5) prior to their responses. The mean (median) number of long COVID symptoms ever experienced by respondents and experienced in the last 30 days were 32 (35) and 18 (13), respectively. The top 10 most experienced symptoms were fatigue (54%), chills (54%), lightheadedness (52%), malaise (52%), dizziness (51%), brain fog (51%), insomnia (50%), headache (50%), physical weakness (50%), myalgia (48%), and tachycardia (48%).

During long COVID, 36 (43%) reported not being able to tolerate any moderate to vigorous exercise, with another 16 (19%) being able to tolerate fewer than 10 min. Notably, of the respondents, 70 (85%) had experienced post-exertional malaise (PEM) at least once during long COVID, with 67 (81%) in the previous 30 days.

**Fig. 1:**
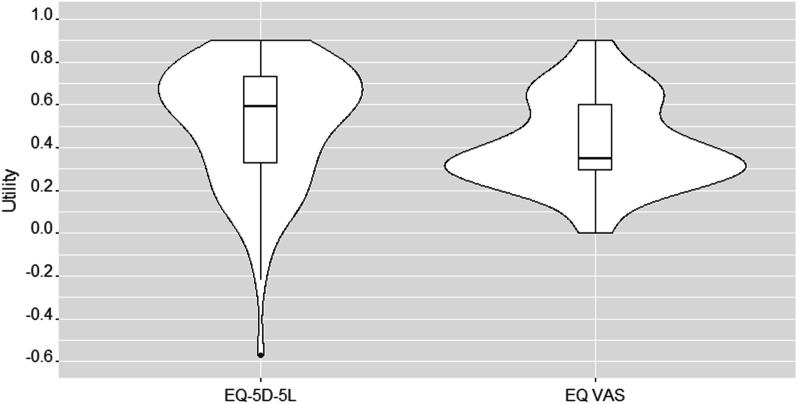
Violin plot of EQ-5D-5L score, all subjects. This violin plot shows the kernal density of the utility scores across the entire sample and a boxplot of the summary scores

**Fig. 2:**
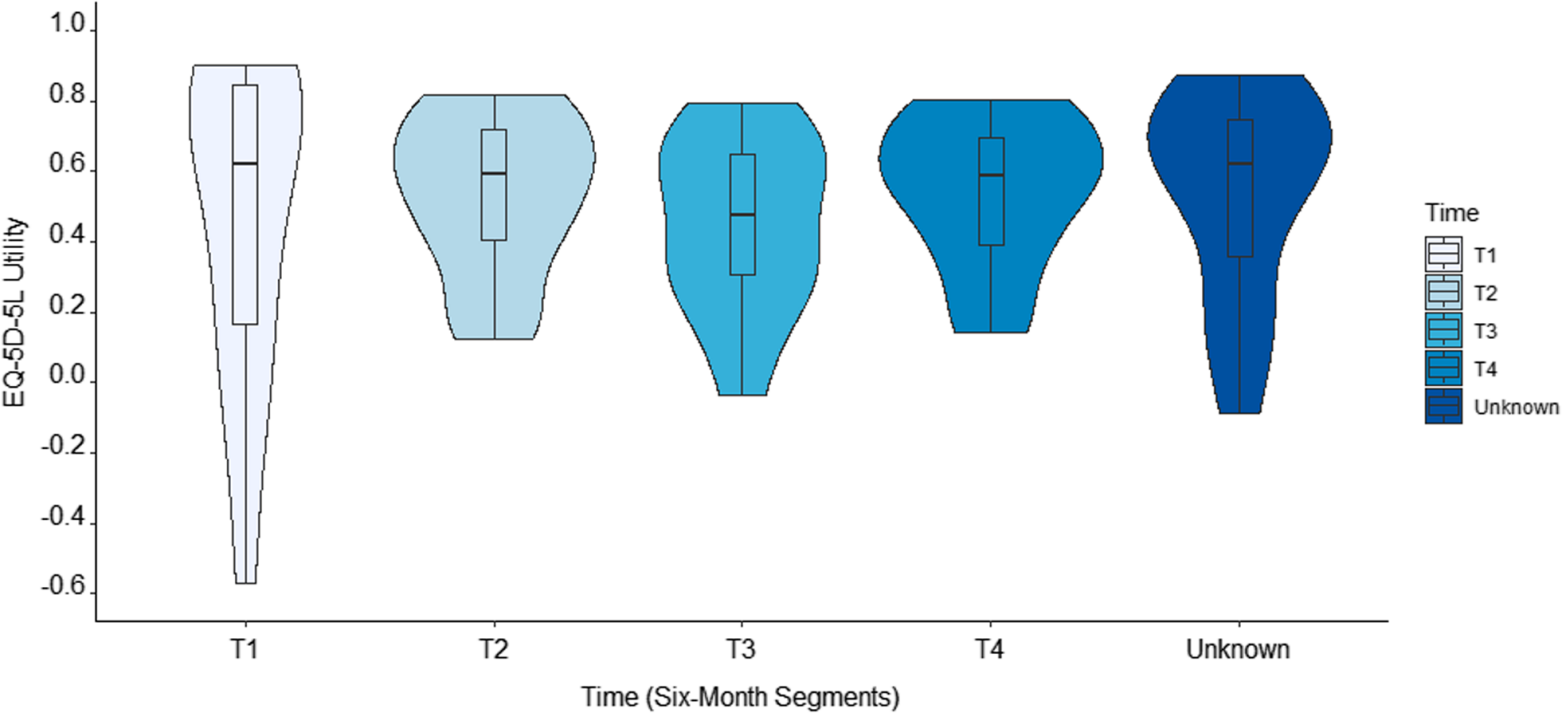
Violin plot of EQ-5D-5L scores, all subjects stratified by time of long COVID onset in six month intervals. These violin plots show the kernal density of the utility scores and a boxplot of the summary scores across the entire sample, broken down by time. T1 = 1–6 Months; T2 = 7–12 Months; T3 = 13–18 Months; T4 = 19 + Months

The mean (median, IQR) EQ-5D-5L utility score was 0.51 (0.59, 0.39) and the EQ VAS was 41.6 (35.0, 31.0, Fig. 1). On average, participants reported the greatest difficulties with usual activities and pain/discomfort (Table 1). Stratified by time intervals, utility scores were highest in the first six months and lowest in the 13- to 18-month timeframe, although these differences were not statistically different (Fig. 2). Across demographic and clinical strata, significantly lower utilities were seen in individuals who are seeking care in a long COVID clinic, who typically exercised at least 5 h per week prior to COVID, and who experienced more symptoms in the previous 30 days. See Table 1 for details.

The mean (median, IQR) WHODAS score was 49.0 (50.0, 27.1). In the previous 30 days, participants reported their symptoms affected them for a mean (median) of 24 (30) days, they were totally unable to carry out usual activities for 15 (12) days, and they cut back or reduced activities for 26 (28) days. The individual items reporting the highest level of disability were difficulties with day-to-day work, being emotionally affected by health problems, and joining in community activities (Table 2). Over time, WHODAS scores were lowest in the first six months and highest in months 13–18, although these findings were not statistically significant. Across demographic and clinical strata, women had significantly higher disability scores as compared to men, individuals with 30+ symptoms had a significantly higher disability score as compared to those experiencing fewer symptoms, and individuals who reported using medications specifically prescribed for long COVID tended to have a higher disability score. See Tables 1 and 2 for details.

**Table 2:** EQ-5D-5L, WHODAS, and PROMIS domains

Domain variable	Mean	SD	Frequency (%)				
			1	2	3	4	5
*EQ-5D-5L*							
Mobility	2.02	0.99	31 (37.4)	26 (31.3)	20 (24.1)	5 (6.0)	1 (1.2)
Self-care	1.65	0.92	49 (59.0)	18 (21.7)	13 (15.7)	2 (2.4)	1 (1.2)
Usual activities	3.32	1.05	3 (3.7)	13 (15.9)	35 (42.7)	17 (20.7)	14 (17.1)
Pain/discomfort	2.73	0.97	10 (12.2)	18 (22.0)	42 (51.2)	8 (9.8)	4 (4.9)
Anxiety/depression	2.46	1.31	23 (28.1)	24 (29.3)	19 (23.2)	6 (7.3)	10 (12.2)
*WHODAS v2.0*							
Standing for long periods	3.18	1.31	10 (13.7)	11 (15.1)	23 (31.5)	14 (19.2)	15 (20.6)
Household responsibilities	3.25	1.00	4 (5.5)	10 (13.7)	30 (41.1)	22 (30.1)	7 (9.6)
Learning a new task	2.73	1.29	15 (20.6)	20 (27.4)	16 (21.9)	14 (19.2)	8 (11.0)
Joining in community activities	3.52	1.00	1 (1.4)	10 (13.7)	26 (35.6)	22 (30.1)	14 (19.2)
Emotionally affected by health problems	3.56	1.00	2 (2.7)	9 (12.3)	20 (27.4)	30 (41.1)	12 (16.4)
Concentrating for ten minutes	2.92	1.11	9 (12.3)	15 (20.6)	28 (38.4)	15 (20.6)	6 (8.2)
Walking a long distance	3.48	1.38	8 (11.0)	10 (13.7)	19 (26.0)	11 (15.1)	25 (34.3)
Washing whole body	1.97	1.11	34 (46.6)	16 (21.9)	16 (21.9)	5 (6.9)	2 (2.7)
Getting dressed	1.77	0.91	36 (49.3)	21 (28.8)	14 (19.2)	1 (1.4)	1 (1.4)
Dealing with people you do not know	2.67	1.25	19 (26.0)	10 (13.7)	25 (34.3)	14 (19.2)	5 (6.9)
Maintaining a friendship	2.68	1.14	13 (17.8)	17 (23.3)	29 (39.7)	8 (11.0)	6 (8.2)
Day to day work	3.78	1.13	1 (1.4)	10 (13.7)	20 (27.4)	15 (20.6)	27 (37.0)
*PROMIS v1.2 Global Health*							
Physical Health Score	10.65	2.85					
Mental Health Score	8.56	3.45					
Health	1.88	0.99	35 (43.8)	27 (33.8)	13 (16.3)	3 (3.8)	2 (2.5)
Quality of life	1.83	0.98	35 (43.8)	32 (40.0)	8 (10.0)	2 (2.5)	3 (3.8)
Physical health	1.89	0.96	34 (42.5)	28 (35.0)	14 (17.5)	2 (2.5)	2 (2.5)
Mental health	2.14	0.97	20 (25.3)	37 (46.8)	16 (20.3)	3 (3.8)	3 (3.8)
Social activities and relationships	1.90	1.06	37 (46.8)	22 (27.9)	13 (16.5)	5 (6.3)	2 (2.5)
Everyday physical activities	3.01	1.18	5 (6.2)	27 (33.3)	24 (29.6)	12 (14.8)	13 (16.1)
Pain	3.23	0.91	1 (1.2)	17 (21.0)	31 (38.3)	26 (32.1)	6 (7.4)
Fatigue	2.57	0.87	8 (9.9)	30 (37.0)	33 (40.7)	9 (11.1)	1 (1.2)
Social activities	1.70	0.89	41 (51.3)	27 (33.8)	8 (10.0)	3 (3.8)	1 (1.3)
Anxiety/depression	2.67	1.28	18 (22.8)	20 (25.3)	18 (22.8)	16 (20.3)	7 (8.9)

The EQ-5D-5L has five domains in which participants rate their problems associated with each of the domains, ranging from no problem (1) to extreme or unable to do (5)

The WHODAS 2.0 12-item assessment asks participants to rate the difficulty they experienced in different areas in the previous 30 days, ranging “none” (1) to “extreme or cannot do” (5)

The PROMIS v1.2 has participants rate their current status in each of these domains. Scores closer to (5) indicate better health

The mean (median, IQR) PROMIS PH and MH scores were 10.7 (10.5, 4.0) and 8.6 (8.0, 4.0), corresponding to mean t-scores of 36.3 (36.3, 10.5) and 34.4 (32.9, 10.8), respectively. Across demographic and clinical strata, significantly lower PH scores, indicating lower physical health, were seen among women, those with a higher 30-day symptom count, and among those seeking care at a long COVID clinic. Significantly lower MH scores were seen among those seeking care at a long COVID clinic. See Tables 1 and 2 for more details.

## Discussion

Long COVID presents a significant chronic health burden to adults globally. In this study, we examined health utility, disability, and health status and found that there were no significant changes over time. We also did not find a monotonic pattern in the 6-month segments, suggesting that the impacts of long COVID may persist for much longer than a year and with no apparent time trend for recovery at the population level. This is concerning considering that many of the respondents were generally healthy prior to contracting COVID, engaged plentifully in exercise, and an overwhelming majority reported mild to moderate acute COVID-19 illness without hospitalization. These results may give insight to providers and policymakers grappling to understand the impact of long COVID on HrQOL, disability, and health status.

We found that individuals experiencing 30 or more symptoms in the previous 30 days had the lowest levels of HrQOL, the highest disability, and the lowest physical health score. This variable likely serves as a proxy for the current severity of their condition. We also found differences between those who had been seen in a long COVID specialized clinical vs those who had not. Generally, those who had been seen in a long COVID clinic had lower health status and QOL as compared to those who had not been seen. This is somewhat reinforced by significantly higher disability scores among those reporting medication use specific to long COVID. These indicators may also serve as proxies for condition severity, as those who are seeking specialized services may have the greatest severity of illness, although additional research would be needed to confirm this.

In this study, we compared the severity of the acute COVID-19 infection with reported HrQOL, disability, and health status. Although the number of respondents were few for asymptomatic and severe categories, we found no statistically significant relationship between the severity of the acute infection and the severity of long COVID, as estimated by the four health metrics. Interestingly, it appeared descriptively that the lowest utilities were among those with asymptomatic and moderate illness. Future research should seek to understand the long-term sequelae of post-acute illness for asymptomatic COVID infection, given 41% of confirmed cases are asymptomatic [19]. Future research should also investigate the etiology of long COVID and its relationship with the acute infection severity.

In this study, respondents reported moderately low health utility scores, equivalent to other health conditions such as fibromyalgia, multiple sclerosis, active herpes zoster infection, and myalgic encephalomyelitis [20–23]. The median health utility in this study was 0.59, compared to a general population estimate of 0.83, although it is unclear what impact the pandemic had on the utility estimates of a general population [24]. Of note, the health utility scores found in this study are slightly lower than those reported by Poudel et al. [7], who reviewed published literature examining HrQOL among patients with long COVID and found that mean utility values ranged from 0.61 to 0.71.

Although we found no significant differences in health utility scores across time, we did find some descriptive trends. First, the variability of scores was greatest in the first six months post infection, with some individual scores dipping below 0 (Fig. 2). This may be due to the wide array of symptoms, ranging from mild to severe, that individuals may experience coupled with novelty of the condition, which may elicit a sense of turmoil and distress [25, 26]. Second, we also found that health utility scores were lowest in the third segment, 13–18 months post long COVID onset (Fig. 2). This may be due to individuals recovering before the 13-to-18-month mark and thus leaving a more severely ill contingent. Alternatively, this could be a time when patients are coming to terms with their chronic illness and the far-reaching impacts it has on their lives [25, 26].

The disability scores found in this study are approximately equivalent to disability scores found in patients with severe rheumatoid arthritis, severe chronic widespread pain, stroke, fibromyalgia and moderate to severe depression [27, 28]. Our median disability score of 50 among individuals with long COVID corresponds to the 95th (94.7) percentile for disability in a general population [11].

The PROMIS PH and MH scores further support significantly lower than average (i.e., “fair”) levels of health. These results indicate that individuals currently experiencing long COVID are suffering debilitating symptoms impacting multiple facets of their life, drawing parallels with the 2003 Severe Acute Respiratory Syndrome (SARS) outbreak including long-term complications/symptoms, reduced physical health and exercise capacity, and low quality of life [29, 30].

The findings described in this study are subject to some notable limitations. The respondents described herein represent a cross-sectional convenience sample of individuals with self-reported long COVID. We were not able to verify COVID-19 laboratory results nor were we able to assess health status in-person. As such, we were unable to confirm previous COVID-19 infections, particularly for those with “suspected” long COVID. However, upon examining the data in a post-hoc analysis, we did find that most individuals with asymptomatic (N = 3/4 [75%]) or mild (N = 27/35 [77%]acute infections, which may be more susceptible to misclassification, indicated a positive COVID-19 or antibody test.

Moreover, we used the definition of long COVID as defined by CDC, which comprises individuals with symptoms persisting for at least four weeks post-COVID infection. The WHO uses a much stricter definition, requiring symptoms at least three months post-infection that last for at least two months. Although it is possible that this sample may include some individuals who may not be eligible according to the WHO definition, we expect this to be minimal as individuals responded that they were, on average, 13 months beyond their acute infection.

By using this convenience sample, it is possible we are misestimating the true health status of the long COVID population. Specifically, we may be overestimating disutility, disability, and underestimating health status as those who have recovered, either partially or fully, may no longer follow these social media support groups or may have not responded to the survey. Moreover, the relatively small sample prevented in-depth examinations of subgroups, such as those with asymptomatic acute infections, or differences in outcomes across countries and regions. The small sample also likely prevented us from statistically detecting clinically significant relationships. For example, the current analysis was powered to detect a change in utility of 0.29. For the largest change we saw across time, 0.14 units, our sample provided 24% power. Although a limitation for this study, the findings herein should provide a starting point for future research investigating these relationships.

Although this study included individuals from 13 countries, these countries were concentrated in North America and Europe with inadequate representation from low- and middle-income countries. Additional investigations are needed to explore these trends and determine their generalizability to all individuals with long COVID.

We used general health assessments in this study. By using general tools, we may be misestimating the true severity of the condition [31]. As future research develops and more tailored tools are available, this should be reevaluated.

Finally, we did not collect information on the management of the acute COVID infection, such as pharmacotherapy use. These are important variables to explore given the relationship that infection severity has with likelihood of long COVID [8].

## Conclusions

Patients with long COVID are experiencing significant disutility, disability, and health impacts for many months after their initial illness. An awareness of the health impacts of long COVID may offer insights to providers and decision-makers as they seek to support individuals with long COVID. Continued efforts should be made to prevent disease transmission and the possible subsequent occurrence of long COVID.

## Supplementary Information

**Additional file 1.** Survey Questionnaire.

## Data Availability

Data used in this study are available upon request.
