# Supplementary material for: The health impact of long COVID: a cross-sectional examination of health-related quality of life, disability, and health status among individuals with self-reported post-acute sequelae of SARS CoV-2 infection at various points of recovery
Source: J Patient Rep Outcomes. 2023 Mar 21;7:31. doi: 10.1186/s41687-023-00572-0 (PMC10029785; doi:10.1186/s41687-023-00572-0)
Supplement: Supplementary file 1 — Additional file 1. Survey Questionnaire. [file 41687_2023_572_MOESM1_ESM.docx]

This study is examining quality of life and health resource utilization for patients with long COVID. We define long COVID, or post COVID-19 condition, as continuing to experience symptoms or health problems, new or existing, due to COVID-19 four weeks and beyond after the initial infection.

- What is today's date?

Before we examine your experience with long COVID, we would like to know a bit about your acute COVID-19 infection (that is, when you first became ill with COVID-19).

The next set of questions will ask you about your acute infection with COVID-19. Please answer to the best of your ability.

- To your knowledge, were you infected with COVID-19?
  - Yes | No | I don't know
- How many times have you been infected?
- If known, approximately when did you first become ill with COVID-19 (either confirmed or suspected)?

If you can't remember, just take your best guess.

- Did you have a positive COVID-19 test (e.g., PCR, rapid/antigen)?
  - Yes | No | I was not tested
- Were you positive for antibodies for COVID-19?
  - Yes | No | I was not tested
- If known, how would you describe your symptoms during the acute COVID-19 infection?
  - Severely ill (i.e., required intensive medical care [e.g., hospitalization]) | Moderately ill (i.e., sought some medical care) | Mildly ill (i.e., managed at home) | Asymptomatic (i.e., no symptoms) | I don't know
- Have you received a COVID-19 vaccine?
  - Yes | No | I don't know | Prefer not to respond
- Which COVID-19 vaccine(s) have you received?
- Approximately when did you receive your FIRST COVID-19 vaccine dose?
- Approximately when did you receive your SECOND COVID-19 vaccine dose?

Now we will look at your experience with long COVID specifically. The Centers for Disease Control and Prevention (CDC) defines long COVID as health problems that "are present four or more weeks after infection" with COVID-19.

The next sets of questions look at which symptoms you have had or are currently experiencing, your current and recent quality of life, and your healthcare utilization.

- Please identify all long COVID related symptoms you have EVER EXPERIENCED since the onset of your long COVID and EXPERIENCED IN THE PAST 30 DAYS.
  Please check all that apply.
  - Fever
  - Chills or shivering
  - Fatigue
  - Physical weakness/lack of energy or asthenia
  - Malaise/feeling sick
  - Extensive sweating or night sweats
  - Decreased sweating
  - Dizziness
  - Brain fog (memory problems/difficulty concentrating)
  - Lightheadedness
  - Vertigo
  - Drowsiness
  - Confusion or delirium
  - Unconsciousness
  - Cough
  - Shortness of breath, dyspnea or respiratory distress
  - Lung expectoration (coughing up phlegm/sputum)
  - Coughing up blood
  - Abnormal oral mucus or saliva
  - Sore throat
  - Throat congestion
  - Tonsil swelling
  - Dry mouth
  - Sneezing
  - Abnormal mucus in nose/nasal congestion
  - Runny nose (rhinorrhea)
  - Nose bleeding
  - Other nasal symptoms
  - Ear pain
  - Hearing loss
  - Tinnitus
  - Ear pressure
  - Eye irritation or sore eyes
  - Red eyes
  - Dry eyes
  - Pink eye (conjunctivitis)
  - Light sensitivity
  - General pain
  - Muscle pain/myalgia
  - Neuropathic/neurological pain
  - Headache
  - Migraine (with or without aura)
  - Muscle soreness
  - Joint pain
  - Neck pain
  - Back pain
  - Shoulder pain
  - Mouth/facial pain
  - Other pain
  - Loss of taste
  - Loss of smell (hyposmia/anosmia)
  - Parosmia (odor distortion) or phantosmia (detecting odors that are not present)
  - Changes in sight/vision problems
  - Uncoordinated movements
  - Seizures
  - Tremors/trembling in arms/legs
  - Muscle twitches/jerks (myclonus)
  - Numbness in arms/legs
  - Other numbness
  - Tingling in arms/legs
  - Other tingling
  - Coldness in arms/legs
  - Diarrhea
  - Constipation
  - Nausea
  - Vomiting
  - Changes in appetite (e.g., loss)
  - Stomachache/abdominal pain
  - Acid reflux/heartburn
  - Abdominal distension (increase in abdominal size)
  - Bloating
  - Tenesmus/Bowel cramps
  - Gastrointestinal discomfort
  - Other gastrointestinal symptoms
  - Dysuria (painful urination)
  - Blood in urine
  - Skin rash
  - Itchy skin
  - Skin pain/burning sensation
  - Hair loss
  - Chest pain
  - Tightness of chest
  - Chest distress
  - Heart palpitations
  - Tachycardia (fast heart rate, over 100 beats per minute)
  - Poor blood circulation
  - Other cardiovascular symptoms
  - Anxiety
  - Depression
  - Tension
  - Anger
  - Agitation
  - Insomnia
  - Other symptom(s)
- Some individuals with long COVID have described experiencing a "relapse" or "flare up" during the course of their illness.
  Have you experienced a "relapse" or "flare up"?
  - Yes, I am currently experiencing one | Yes, I have experienced at least one previously | No | I don't know
- How often do you experience relapses or flare ups?
  - Several times per week | About once per week | About once per month | Less than once per month | It varies | I don't know
- How long do your relapses typically last?
  - A few hours | More than a few hours but less than one day | One to two days | More than two days but less than one week | One to two weeks | More than two weeks
- Please click the ONE box that best describes your health TODAY.
  - Mobility
    - I have no problems walking | I have slight problems walking | I have moderate problems walking | I have severe problems walking | I am unable to walk
  - Self-Care
    - I have no problems washing or dressing myself | I have slight problems washing or dressing myself | I have moderate problems washing or dressing myself | I have severe problems washing or dressing myself | I am unable to wash or dress myself
  - Usual Activities (e.g., work, study, housework, family or leisure activities)
    - I have no problems doing my usual activities | I have slight problems doing my usual activities | I have moderate problems doing my usual activities | I have severe problems doing my usual activities | I am unable to do my usual activities
  - Pain/Discomfort
    - I have no pain or discomfort | I have slight pain or discomfort | I have moderate pain or discomfort | I have severe pain or discomfort | I have extreme pain or discomfort
  - Anxiety/Depression
    - I am not anxious or depressed | I am slightly anxious or depressed | I am moderately anxious or depressed | I am severely anxious or depressed | I am extremely anxious or depressed
- We would like to know how good or bad your health is TODAY.
  This scale is numbered from 0 to 100.
  100 means the best health you can imagine.
  0 means the worst health you can imagine.
  Please click on the scale to indicate how your health is TODAY.
- In general, would you say your health is:
  - Excellent | Very Good | Good | Fair | Poor
- In general, would you say your quality of life is:
  - Excellent | Very Good | Good | Fair | Poor
- In general, how would you rate your physical health?
  - Excellent | Very Good | Good | Fair | Poor
- In general, how would you rate your mental health, including your mood and your ability to think?
  - Excellent | Very Good | Good | Fair | Poor
- In general, how would you rate your satisfaction with your social activities and relationships?
  - Excellent | Very Good | Good | Fair | Poor
- In general, please rate how well you carry out your usual social activities and roles. (This includes activities at home, at work and in your community, and responsibilities as a parent, child, spouse, employee, friend, etc.).
  - Excellent | Very Good | Good | Fair | Poor
- To what extent are you able to carry out your everyday physical activities such as walking, climbing stairs, carrying groceries, or moving a chair?
  - Completely | Mostly | Moderately | A little | Not at all
- In the past 7 days, how often have you been bothered by emotional problems such as feeling anxious, depressed or irritable?
  - Never | Rarely | Sometimes | Often | Always
- In the past 7 days, how would you rate your fatigue on average?
  - None | Mild | Moderate | Severe | Very Severe
- In the past 7 days, how would you rate your pain on average?
  - 0, No Pain | 10, Worst Pain Imaginable
- Think back over the past 30 days and answer these questions, thinking about how much difficulty you
  had doing the following activities. For each question, please mark only one response.
  In the past 30 days, how much difficulty did you have in....
  - Standing for long periods such as 30 minutes?
    - None | Mild | Moderate | Severe | Extreme or cannot do
  - Taking care of your household responsibilities?
    - None | Mild | Moderate | Severe | Extreme or cannot do
  - Learning a new task, for example, learning how to get to a new place?
    - None | Mild | Moderate | Severe | Extreme or cannot do
  - How much of a problem did you have joining in community activities (for example, festivities, religious or other activities) in the same way as anyone else can?
    - None | Mild | Moderate | Severe | Extreme or cannot do
  - How much have you been emotionally affected by your health problems?
    - None | Mild | Moderate | Severe | Extreme or cannot do
  - Concentrating on doing something for ten minutes?
    - None | Mild | Moderate | Severe | Extreme or cannot do
  - Walking a long distance such as a mile (or kilometer)?
    - None | Mild | Moderate | Severe | Extreme or cannot do
  - Washing your whole body?
    - None | Mild | Moderate | Severe | Extreme or cannot do
  - Getting dressed?
    - None | Mild | Moderate | Severe | Extreme or cannot do
  - Dealing with people you do not know?
    - None | Mild | Moderate | Severe | Extreme or cannot do
  - Maintaining a friendship
    - None | Mild | Moderate | Severe | Extreme or cannot do
  - Your day-to-day work?
    - None | Mild | Moderate | Severe | Extreme or cannot do
- "Overall, in the past 30 days, how many days were these difficulties (from the last question) present?
- In the past 30 days, for how many days were you totally unable to carry out your usual activities or work because of any health condition?
- In the past 30 days, not counting the days that you were totally unable, for how many days did you cut back or reduce your usual activities or work because of any health condition?

We would like to ask you about your health care use.

- Have you received care at a specialized long COVID clinic?
  - Yes | No | I don't know
- How many visits have you attended at the long COVID clinic? Please include any telemedicine visits.
- Are you taking any medications or supplements specific to managing long COVID?
  - Yes | No | I don't know

Demographics and Health Behaviors

- PRIOR to long COVID, how many MINUTES PER WEEK did you engage in MODERATE or VIGOROUS exercise?

Examples of moderate exercise are a brisk walk or yoga.

Examples of vigorous exercise are jogging or running.

- Have you experienced post-exertional malaise (PEM) after any activity (e.g., physical, mental) during long COVID?
  PEM is defined by the Centers for Disease Control and Prevention (CDC) as "the worsening of symptoms following even minor physical or mental exertion, with symptoms typically worsening 12 to 48 hours after activity and lasting for days or even weeks.”
  - Yes | No | I don't know
- In the PAST 30 DAYS, have you experienced post-exertional malaise (PEM) after any activity (e.g., physical, mental) during long COVID?
  PEM is defined by the Centers for Disease Control and Prevention (CDC) as "the worsening of symptoms following even minor physical or mental exertion, with symptoms typically worsening 12 to 48 hours after activity and lasting for days or even weeks.”
  - Yes | No | I don't know
- What is your height in inches?
- What is your weight in pounds?
- What is your age in years?
- What is your gender?
  - Man | Woman | Transgender Man | Transgender Woman | Non-binary | Other | I prefer not to respond
- What is the highest degree or level of education that you have completed?
  - Less than high school, no diploma | High school graduate, diploma or the equivalent (for example: GED) | Some college credit, no degree | Trade/technical/vocational training | Associate degree (for example: AA, AS) | Bachelor's degree (for example: BA, BS) | Master's degree (for example: MA, MS, MEng, MEd, MSW, MBA) | Doctorate or Professional degree (for example: PhD, MD, DO, JD, EdD, LLB, DDS, DVM, PsyD, PharmD)
- Which forms of health insurance do you have? Choose all that apply.
  - Private health insurance | Government-sponsored health insurance (including national programs) | Other health insurance | I don't have health insurance | I don't know
- What is your current employment status?
  - Employed full-time | Employed part-time | Self-employed/Freelance | Interning | Unemployed- Looking for work | Unemployed - Not looking for work | Student | Military/Armed Forces | Retired | Not able to work | Other
- Which race(s) do you identify with?
  Check all that apply.
  - White | Black/African American | American Indian or Alaska Native | Asian | Pacific Islander | Other | I prefer not to respond
- Do you consider yourself Hispanic/Latino?
  - Yes | No | I don't know
- Which country are you living in?
- How would you describe your residential environment?
  - Urban | Suburban | Rural | I don't know
